# Adaptation of digital integration of PROMs and PREMs in oncology during implementation: a scoping review

**DOI:** 10.1007/s00520-026-10509-0

**Published:** 2026-03-13

**Authors:** Nuša Farič, Anne-Lore Scherrens, Eveline Raemdonck, Kim Beernaert, Kathrin Cresswell, An Jacobs, Tonje Lundeby, Koen Pardon, Robin Williams, Femke Van Landschoot, Judith de Vos-Geelen, Dag Ausen, Amaia Urrizola, Marianne Jensen Hjermstad, Stein Kaasa, Marie Fallon, Luc Deliens, MyPath consortium, Kate Absolom, Morten Andresen, Marek Atter, Dag Ausen, Sara Bea, Kim Beernaert, Augusto Caraceni, Andres Cervantes, Kathrin Cresswell, Olav Dajani, Judith de Vos-Geelen, Luc Deliens, Felicity Evans, Marie Fallon, Victòria Freitas-Durks, Viviana Fusetti, Inez Gonzalez-Barrallo, Peter Hall, Marianne Jensen Hjermstad, Marisol Huerta, Kristin Solheim Hustad, An Jacobs, Stein Kaasa, Lisa Heide Koteng, Geana Paula Kurita, Henrik Larsen, Ulrik Lassen, Nicola Jane Latino, Tonje Lundeby, Elias David Lundereng, Camilla Charlotte Lykke, Giacomo Massa, Ulla Mathiesen, Nicoleta Mitrea, Daniela Mosoiu, Steven Olde Damink, Helle Pappot, Koen Pardon, Cathy Payne, Oana Predoiu, Anne-Lore Scherrens, Morena Shkodra, Per Sjøgren, Eivind Storaas, Amaia Urrizola, Peder Heyderdahl Utne, Femke Van Landschoot, Galina Velikova, Lorraine Warrington, Naomi White, Robin Williams

**Affiliations:** 1 Institute for Adaptive and Neural Computation, School of Informatics, The University of Edinburgh, 10 Crichton St, Edinburgh, EH8 9AB UK; 2 Advanced Care Research Center (ACRC), The University of Edinburgh, Edinburgh, UK; 3 End-of-Life Care Research Group, Vrije Universiteit Brussel (VUB) and Ghent University, Brussels, Belgium; 4 Usher Institute, The University of Edinburgh, Edinburgh, UK; 5 Imec-SMIT Research Group, Department of Media and Communication Studies, Vrije Universiteit Brussel, Brussels, Belgium; 6 Institute of Clinical Medicine, University Hospital, University of Oslo, Oslo, Norway; 7 Institute for the Study of Science, Technology and Innovation, The University of Edinburgh, Edinburgh, UK; 8 Department of Internal Medicine, Division of Medical Oncology, GROW - Research Institute for Oncology & Reproduction, Maastricht University Medical Center, Maastricht, the Netherlands; 9 DNV Imatis, Porsgrunn, Norway; 10 Edinburgh Cancer Research Centre, Institute of Genetics and Cancer (IGC), The University of Edinburgh, Edinburgh, UK; 11 SMIT, Vrije Universiteit Brussel (VUB), Brussels, Belgium; 12 Health Promotion Unit, Ghent University, Ghent, Belgium

**Keywords:** Palliative care, Patient-reported outcome measures, Digital health, Psycho-oncology, Care management–patient, Healthcare quality indicators

## Abstract

**Purpose:**

Digital tools facilitate the timely collection of patient-reported outcome and experience measures (ePROMs/PREMs), but there is no consistent reporting on the technical and content adaptations made essential to implementing these digital tools in a specific context. Adaptations made to ePROMs/ePREMs can improve data quality, clinical management, and patient outcomes. We explored how studies report on adaptations and the reasons and types of these during an implementation process of ePROMs/ePREMs systems in routine cancer care.

**Methods:**

We conducted a systematic scoping review. We searched PubMed, Embase, PsychINFO, and CINAHL (inception—May 5, 2023), using the Preferred Reporting Items for Systematic Reviews and Meta-Analyses extension for Scoping Reviews (PRISMA-ScR) checklist. Guided by the Population, Concept, and Context (PCC) framework, data were extracted and summarised in tables in four dimensions: context, content, evaluation, and training.

**Results:**

The systematic search found *n* = 5597 publications, and *n* = 20 were included (85% published since 2019). No studies reported on ePREMs. Various data collection methods and stakeholders were utilised to make adaptations, guided by one or more implementation frameworks (80% of studies). Common types of adaptations included changing context (e.g. complex onboarding), content (e.g. readability) (all studies), evaluation (e.g. alerts), and training (e.g. patients and clinicians). The use of an implementation framework did not affect the types of adaptations made.

**Conclusions:**

This review summarises the types of adaptations made to oncology ePROMs during implementation. To date, there has been no agreed system to capture adaptations of ePROMs in oncology, nor a system or framework to assess ePROMs efficacy.

**Supplementary Information:**

The online version contains supplementary material available at 10.1007/s00520-026-10509-0.

## Relevance Statement

i) What is already known about the topic?
  To date, there has been no agreed system to capture adaptations of ePROMs in oncology, and currently, there is no system or framework to assess ePROMs’ efficacy.
  This review offers a complete summary description of the reasons and types of adaptations made to oncology ePROMs during an implementation process.

ii) What this paper adds
  We created a complete overview of reasons and types of adaptations made to ePROMs which can be used in future development and implementation of ePROMs in oncology evidence.

iii) Implications for practice, theory or policy
  Our review showed the complete absence of studies that include adaptations to oncology ePREMs, potentially indicating a gap in reporting on patients’ experiences, while much focus has been on patient outcomes (ePROMs).
  Our review highlighted the low availability of data for in-patient groups and these patient groups could benefit from being included more frequently in the literature.
  Our review also found no relationship between the type of an implementation framework and adaptation made to ePROMs

## Introduction

In recent years, health information technology (HIT) has become an integral part of oncology care to facilitate the electronic assessment of patient-reported outcome measures (PROMs) and patient-reported experience measures (PREMs) [1, 2]. Electronic PROMs (ePROMs) assess patients’ views on their health, well-being, or symptoms related to their care (e.g. nausea and pain). ePREMs capture patients’ perspectives on the overall quality of and satisfaction with received care, which is essential for personalised oncology treatment and holistic care [3]. Assessing ePROMs and ePREMs can help to identify unmet patient needs, improve patient-clinician communication, patient satisfaction, increase supportive care measures, alleviate symptom burden, and improve patient-centred care [4–7]. Real-world implementation of ePROMs, and in particular ePREMs, outside the context of feasibility or research intervention in cancer care remains sparse, and adoption/implementation attempts rates are low [2, 8, 9].

Up to now, the scientific literature shows that ePROMs and ePREMs are prone to different barriers at distinct stages of the implementation process in healthcare [10–13]. These include the following: lack of clinicians’ knowledge; disbelief in their value; lack of digital literacy of both patients and clinicians; technology confidence; inadequate infrastructures; and challenges with integrating ePROMs and ePREMs into the electronic medical health record [2, 10]. Adaptations are an integral part of the implementation process of ePROMs and ePREMs. Adaptations refer to a process of purposeful or unplanned modifications to system design or implementation to fit the local context better, to retain fidelity (maintaining the integrity of the intervention and its implementation), or to facilitate acceptability [14–17]. Many early-stage implementation reports provide preliminary observations rather than established findings. We aimed to base the review on evidence derived from fully executed interventions to avoid drawing conclusions based on incomplete or tentative data. In the ePROMs/ePREMs literature, most studies do not track nor report on the adaptations performed. Adaptations to ePROMs/ePREMs in oncology should be a topic of research because they directly impact data quality, clinical decision-making, and patient engagement. ePROMs/PREMs must be tailored to the specific context to be successfully integrated, as there is no one-size-fits-all solution. Consequently, ongoing formative evaluation and adaptation are essential strategies. Optimised electronic measures can play an important role in early detection of complications and tailoring of patients’ disease management, potentially improving quality of life or survival [5].

In this scoping review, therefore, we try to map those studies that describe ePROMs and ePREMs adaptations and map how and what types of adaptations are reported to try to improve fit with the local context during the implementation study. Learning about adaptations made to ePROMs/ePREMs in oncology could facilitate informing the development of other systems or outcome measures applied in oncology.

## Methods

### Design

The Preferred Reporting Items for Systematic reviews and Meta-Analyses extension for Scoping Reviews (PRISMA-ScR) checklist and explanation [18] was used to draft the protocol which was subsequently registered with the Open Science Framework 2022 https://osf.io/z3wc7/overview. A systematic scoping review methodology was chosen as it was most appropriate to the study aim of mapping the existing literature to provide a more exploratory overview of a topic that has not been previously characterised in this manner.

### Eligibility criteria

To be included in this review, articles needed to (i) focus on routine cancer care, (ii) report on the implementation of an ePROMs and/or ePREMs intervention, and (iii) (retrospectively) describe an iterative process of adapting the system and/or methods of implementation. As adaptations often take place after the initial stages of the ePROMs/ePREMs implementation, it is therefore important that studies describing complete implementations were identified. RCTs are not designed to assess the sustainability of implementing ePROMs in daily practice and often do not allow for the adaptations needed for real-world use—both important elements of our scoping review. Articles were excluded if they did not/were (i) not oncology focussed; (ii) implemented paper-based PROMs and PREMs; (iii) exclusively focused on the impact, effectiveness, measurement properties, and/or language validity of ePROMs and ePREMs; (iv) used ePROMs and ePREMs as a measure of therapy effect of drugs, such as in clinical trials; and (v) described the implementation of ePROMs and ePREMs in research contexts (e.g. clinical trials) or were prospective because these were usually still ongoing. Furthermore, the search was limited to primary studies written in English. Duplicates, protocols, non-empirical publications, editorials, letters to editors, conference abstracts, and commentaries were also excluded. Reviews were not included in the synthesis, but reference lists of relevant reviews were screened for primary studies. Full criteria are available in the Supplementary Material 01.

### Literature search

We systematically searched the following academic databases: PubMed, Embase, PsychINFO, and CINAHL (from inception to May 5, 2023). Through multidisciplinary team discussion, a search strategy was developed combining indexing terms, keywords, and synonyms for cancer (e.g. neoplasm), implementation and adaptation (e.g. adoption), and PROMs/PREMs (e.g. patient-reported outcomes). The full search strategy was adapted for each electronic database (see Supplementary Material 01). All records were imported using the reference manager EndNote (Clarivate), and the screening process was facilitated by the web-based tool Rayyan (Rayyan Systems Inc.). EndNote allowed us to import the references more quickly than downloading each reference individually and importing it into Rayyan. After removing duplicates, records were screened for eligibility based on title and abstract by E.R. (see Acknowledgements). In this first round of screening (i.e. based on title and abstract), a second reviewer (N.F.) independently screened at least 10% (*n* = 494) to check inter-rater reliability. This was followed by a screening on full-text level, in which all records were screened by the two reviewers (E.R. and N.F.) blinded to each other. Disagreements and conflicts were resolved through discussion. The electronic database search was supplemented by screening reference lists of relevant reviews and included papers, by exploring grey literature (the last search was on July 24, 2023), all in the English language. The latter included exploring Dutch literature (in the English language) in Medline, MedRxiv (i.e. preprints), the International Network of Agencies for Health Technology Assessment (HTA) database, and Open Access Theses and Dissertations. We used this because a significant proportion of the authors on our paper are based in the Dutch-speaking part of Belgium.

### Data extraction and synthesis

This review was guided by the Population, Concept, and Context (PCC) framework [14], which helped us construct clear and meaningful objectives and categories for data extraction in our scoping review. The data from the included studies were extracted and summarised in a table in Excel (Microsoft Inc) by one researcher (E.R.), and uncertainties were discussed with a second (N.F.) and third reviewer (A.-L.S.). The extracted data included PCC dimensions: context, content, evaluation, and training—categories that shaped our data organisation and discussion.

The extracted data also included (i) background information: first author, year, and country where the implementation took place; (ii) details on the population and setting: number of sites where the implementation took place, in- and/or outpatient setting, and target group; (iii) details on the ePROMs and/or ePREMs intervention: measurement instruments, timing, and core features of the digital system (e.g. alert system and integration in the electronic medical health record); (iv) information on the process and reporting of adaptation: theoretical frameworks, process for making adaptations, clinical champions supporting the adaptation process, qualitative and/or quantitative methods supporting the adaptation process (methodology described for the adaptation), and presentation of adapted elements in the manuscript (i.e. first aim); and (v) reasons for and types of adaptations (i.e., second aim). Stirman and associates’ typology of modifications was used to categorise adaptations [14]. Contextual adaptations refer to changes to the delivery of the ePROMs/ePREMs in terms of format, setting, personnel, and the target population, while content adaptations refer to changes to the procedures, materials, or delivery. Adapting training and evaluation processes refer to training and evaluation adaptations, respectively [14]. Numerical analyses using descriptive methods and a narrative synthesis were conducted to summarise results.

## Results

### Study selection

**Fig. 1:**
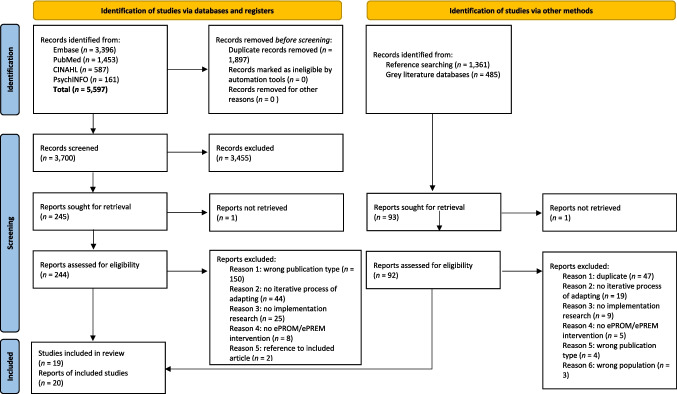
PRISMA-ScR flow diagram

As shown in the PRISMA-ScR flow diagram [18] (Fig. 1), the systematic search in academic databases resulted in *n* = 5597 publications. The titles and abstracts of 3700 records were screened for eligibility after the removal of duplicates. From these, 244 were retrieved for the full-text screening round. The full-text screening round identified 15 studies eligible for inclusion. The main reasons for exclusion were the wrong publication type (i.e. conference abstracts and literature reviews) and the lack of an iterative process of adapting the system and/or implementation methods. Per cent agreement between the first and second reviewers was 92.5% and 94.7% in the first (i.e. title and abstract) and second (i.e. full text) screening rounds, respectively. Scanning reference lists of relevant reviews resulted in five additional studies being identified. We did not find grey literature publications eligible for inclusion. In total, *n* = 20 articles were included in this scoping review, in which two publications reported on the same ePROM intervention and the accompanying process of adapting the digital tool [19, 20].

### General study characteristics

Among the included studies, the oldest one dated from 2010 [21], and *n* = 17 (85%) of the studies were published between 2019 and 2022, with eight studies alone in 2022 [16, 19, 20, 22–25]. Studies reported locations in which they were conducted as follows: USA (*n* = 7) [16, 22, 25–29], Europe (*n* = 6; i.e. Austria (*n* = 3), Germany, Belgium, and the Netherlands) [21, 23, 24, 30–32], Australia (*n* = 3) [19, 20, 33, 34], and the UK (*n* = 2) [35, 36]. In one study, the implementation took place in different countries [37]. Concerning the target group, *n* = 15 (70%) targeted outpatients [19–21, 23, 26–31, 33–37] and *n* = 12 (60%) included patients with various cancer diagnoses [16, 21, 24–27, 29, 32, 33, 37] (Table 1). Implementation of ePROMs assessments was guided by various groups such as study authors, clinicians, administration staff, champions, nurses, etc. (see under “Process of integration and adaptations”). Two studies included children with cancer [21, 26].

**Table 1:** Study characteristics

First author, year	Country	Number of centres^b^	In- and/or outpatients	Types of cancer(s)^d^
Bamgboje‐Ayodele, 2022 [20]^a^ Girgis, 2022 [19]^a^	Australia	3	Outpatients	Lung cancer
Engelen, 2010 [21]	Netherlands	4	Outpatients	Children
Fernández-Méndez, 2018 [35]	UK	2	Outpatients	Brain tumour or metastasis
Furlong, 2019 [37]	Norway; Austria; Greece; Ireland; UK	13	Outpatients	Breast cancer, colorectal cancer, Hodgkin’s disease, or non-Hodgkin’s lymphoma
Hassett, 2022 [22]	USA	6	In- and outpatients	Gynecologic, thoracic, and GI malignancies
McClaery, 2022 [26]	USA	1	Outpatients	Children and adults diagnosed with cancer or blood disorders
Misplon, 2022 [23]	Belgium	1	Outpatients	Lung cancer
Neal, 2021 [27]	USA	2	Outpatients	Breast and gastrointestinal (GI)
Nordhausen, 2022 [24]	Germany	1	Inpatients	Radiation oncology patients
Palos, 2021 [28]	USA	4	Outpatients	All cancer types
Roberts, 2020 [33]	Australia	2	Outpatients	A wide range of cancer types, care needs, and level of complexity in children/pediatric oncology
Rocque, 2022 [16]	USA	2	In- and outpatients	All cancer types
Schuler, 2021 [34]	Australia	1	Outpatients	Lung, prostate, colorectal, breast and other
Strachna, 2022 [25]	USA	20^c^	In- and outpatients	All cancer types
Sztankay, 2019 [30]	Austria	1	Outpatients	Multiple myeloma
Van Egdom, 2019 [31]	Netherlands	1	Outpatients	Breast cancer
Warrington, 2019 [36]	UK	1	Outpatients	Early breast cancer
Wintner, 2020 [32]	Austria	1	Inpatients	Various diagnoses
Wu, 2016 [29]	USA	1	Outpatients	Breast and prostate cancer

^a^Reporting on the same ePROM system

^b^Centres refer to any hospitals, clinics, or care wards

^c^Approximately 20 sites

^d^The cancer types are reported in line with the wording in the original papers

### Intervention characteristics

All the included studies focused on ePROMs, and none reported on ePREMs. A variety of measurement instruments were used to assess PROMs, including European Organisation for Research and Treatment of Cancer Quality of Life Questionnaire Core (EORTC QLQ) and its specific modules (*n* = 7) [23, 24, 29–32, 35], Patient-Reported Outcomes version of the Common Terminology Criteria for Adverse Events (PRO-CTCATE) (*n* = 5) [16, 22, 23, 33, 36], EQ-5D (health-related quality of life) and its three-level version (*n* = 3) [30–32], and Patient-Reported Outcome Measurement Information System (PROMIS) (*n* = 2) [27, 29] (Table 2). The frequency and timing points of the ePROMs assessment varied ranging from daily (*n* = 2) [24, 37], weekly (*n* = 3) [16, 23, 36], monthly (*n* = 3) [19, 20, 27] to yearly (*n* = 1) [26]. More than half of the studies reported that the frequency of ePROM assessment was either varied or very specific to their study (e.g., at a new cancer diagnosis, prior treatment, depending on treatment, and before a consultation) (*n* = 11) [21, 22, 28, 29, 31, 32, 35]. In terms of the access location, the sites offered either remote, in situ, or a combination of both as follows: in situ (*n* = 5) [21, 24, 30, 33, 35], remote (*n* = 7) [26, 31, 36, 38–41], and both (in situ and remote) (*n* = 7) [19, 20, 22, 26, 27, 29, 32, 34]. In addition, 11 ePROM systems offered alerts [16, 19, 20, 22, 23, 25, 28, 29, 33, 36, 37] and *n* = 14 (64%) studies [16, 19, 20, 22, 25, 29, 31, 33] reported integration of patients’ ePROMs into the electronic health record (EHR).

**Table 2:** Intervention characteristics

First author, year	PROMs Measurement instrument and additional questions	Frequency or timing points	Access location	Alert system	Integration into EHR
Bamgboje‐Ayodele, 2022 [20]; Girgis, 2022 [19]	ESAS; DT; Problem checklist	Monthly	In situ and remote	Yes	Yes
Engelen, 2010 [21]	PedsQL; TAPQOL	Three consecutive consultations	In situ	Unknown	Unknown
Fernández-Méndez, 2018 [35]	EORTC QLQ-C30; EORTC QLQ-BN20	Prior to consultation	In situ	Unknown	Unknown
Furlong, 2019 [37]	Chemotherapy Toxicity Self-Assessment Questionnaire	Daily	Remote	Yes	unknown
Hassett, 2022 [22]	PRO-CTCAE; pictogram questions for overall well-being and functional status	Depending on treatment	In situ and remote	Yes	Yes
McClaery, 2022 [26]	e-NPIQ	Yearly or with new cancer diagnosis	In situ and remote	Unknown	Yes
Misplon, 2022 [23]	PRO-CTCAE; questions on psychological, spiritual, palliative, social, family-related and financial needs; EORTC QLQ-C30; EORTC QLQ-LC13	Weekly	Remote	Yes	Yes
Neal, 2021 [27]	PROMIS; question to request additional help; Faith and Belief, Importance, Community, Address in Care spiritual health questions; open-ended question to add symptoms	Monthly or less frequently (three days in advance of a scheduled visit)	In situ and remote	Unknown	Yes
Nordhausen, 2022 [24]	EORTC QLQ-C30; QSC-R10; three-eight questions for specific symptoms for lung, head-neck, colorectal, brain, breast, and prostate cancer	Daily	In situ	Unknown	Yes
Palos, 2021 [28]	MDASI	Depending on site	Remote	Yes	Yes
Roberts, 2020 [33]	PRO-CTCAE	Prior to consultation	In situ	Yes	Yes
Rocque, 2022 [16]	PRO-CTCAE	Weekly	Remote	Yes	Yes
Schuler, 2021 [34]	NPRS	At baseline and four weeks post therapy	In situ and remote	Unknown	Unknown
Strachna, 2022 [25]	Acute Side Effects From Breast Radiation; COVID-19 Symptom Questionnaire; Daily Symptom Assessment; Recovery Tracker	Varying	Remote	Yes	Yes
Sztankay, 2019 [30]	EORTC QLQ-C30; EORTC QLQ‐MY20; EQ‐5D‐5L	At any given post‐diagnostic time point (according to treatment schedule)	In situ	Unknown	Unknown
Van Egdom, 2019 [31]	EORTC QLQ-C30; EORTC QLQ‐BR23; BREAST-Q; EQ‐5D‐5L; DT; RCS-NL; CarerQoL-7D	Three weeks before the scheduled consultation, following the last course of therapy, six months after surgery and annually thereafter	Remote	Unknown	Yes
Warrington, 2019 [36]	PRO-CTCAE; option to add symptoms; free text option	Weekly or more frequently	Remote	Yes	Yes
Wintner, 2020 [32]	EORTC QLQ-C30; HADS-D; SSS-PSD; EQ-5D-3L; four questions assessing patients’ wish for a psycho-oncology treatment, previous psychiatric disorder and/or psychotherapeutic treatment	Before admission and up to three days before discharge	In situ and remote	Unknown	Yes
Wu, 2016 [29]	EORTC QLQ-C30; Supportive Care Needs Survey-short Form; PROMIS-short form	Prior to consultation	In situ and remote	Yes	Yes

*PRO-CTCAE* Patient-Reported Outcomes version of the Common Terminology Criteria for Adverse Events, *EHR* electronic health record, *EORTC QLQ-C30* European Organisation for Research and Treatment of Cancer Quality of Life Questionnaire Core 30, *HADS-D* Hospital Anxiety and Depression Scale, *SSS-PS* Short Screening Scale for DSM-IV Post-Traumatic Stress Disorder, *EQ-5D-3L* EuroQOL-5-Dimensions, *QSC-R10* Questionnaire on Stress in Cancer Patients, *ESAS* Edmonton Symptom Assessment Scale, *DT* distress thermometer, *e-NPIQ* Electronic New Patient Intake Questionnaire, *PROMIS* Patient-Reported Outcomes Measurement Information System questionnaire, *MDASI* MD Anderson Symptom Inventory, *EORTC QLQ-BN20* European Organisation for Research and Treatment of Cancer Quality of Life Questionnaire Brain Tumor Module, *EORTC QLQ-LC13* European Organisation for Research and Treatment of Cancer Quality of Life Questionnaire Lung Cancer Module, *EORTC QLQ‐MY20* European Organisation for Research and Treatment of Cancer Quality of Life Questionnaire Myeloma Module, *EORTC QLQ‐BR23* European Organisation for Research and Treatment of Cancer Quality of Life Questionnaire Lung Cancer Module, *EORTC QLQ‐MY20* European Organisation for Research and Treatment of Cancer Quality of Life Questionnaire Breast Cancer Module, *RCS-NL* Reproductive Concerns Scale, *CarerQoL-7D* Care-related Quality of Life instrument, *PedsQL* Pediatric Quality of Life Inventory, *TAPQOL* TNO-AZL Preschool Children Quality of Life, *NPRS* Numerical Pain Rating Scale

### Types of adaptations

As shown in Table 3, studies included different types of adaptations, which were categorised into four dimensions. For complete details and descriptions of each type of adaptation, please refer to the table. Types of adaptations included context adaptations (e.g. increasing visibility of the tool in services, changing complex onboarding) (*n* = 12) [16, 19, 20, 27, 30, 32, 33, 35] and content adaptations (e.g. changing visibility, readability, and feedback within the ePROMs system) [16, 19–37] which were included in all 20 studies. Other adaptations were made to the evaluation (e.g. mostly configuration of alerts) (*n* = 3) [22, 33, 35] and training (e.g., group training, training new staff) (*n* = 10) [19–22, 26, 28, 33–37] (see also Supplementary Material 02 for an overview).

**Table 3:** Adaptation types

First author, year	Adaptation type			
	1. Context example	2. Content example	3. Evaluation example	4. Training example
Bamgboje‐Ayodele, 2022 [20]; Girgis, 2022 [19]	Difficulty in identifying whether patients were missed in the onboarding process, which resulted in the refinement of the criteria for patient eligibility	Data required to measure implementation is unavailable, which resulted in integrating reporting measures as part of the implementation workflow to measure implementation	Limited insights into the changes in readiness for, and views on, implementing PROMs as routine care, which resulted in surveys to assess organisational readiness for change and health care professionals’ views on the conducted PROMs implementation	New staff joining the cancer service with no knowledge of PROMs, which resulted in orientations to train new staff
Engelen, 2010 [21]		Feelings of triumph or disappointment when children’s score was over or under the red dotted line, which resulted in parents and children receiving PROMs without line graphs		The theoretical part was not as useful as the practical component of the training, which resulted in the group training being shortened to one hour, and the theoretical part was limited
Fernández-Méndez, 2018 [35]	Progressive departure of the support team from the neuro-oncology clinic, which resulted in rearranging the roles in the implementation	Lack of clarity of PROMs answers, which resulted in including graph bars to summarise patient answers	Insufficient time for members of the care team to prepare PROMs during/before clinic, which resulted in weekly monitoring of PROMs preparation	Members of the care team sometimes forget how to implement the PROMs, which resulted in guidelines with detailed descriptions of how to access and administer the PROMs during clinic
Furlong, 2019 [37]	Inefficient charging (one handset), which resulted in two active handsets being necessary at each cancer centre, with one in use and the second on charge	A four-hour response timeframe was not feasible in busy cancer centres, which resulted in the timeframe for handling mild to moderate patient symptoms being changed from four to eight hours		Technical issues in relation to using the clinician website, which resulted in the technology partner providing additional training on using the system
Hassett, 2022 [22]	Limited access to internet-enabled devices and technology confidence, which resulted in the implementation of alternative PROMs collection methods, including tablets in the clinic and proxy reporting at home	The report displayed all reported symptoms rather than summarising just the severe ones, which resulted in severe symptoms being highlighted in yellow to make them stand out		No encouragement from the care team to submit symptom reports, which resulted in additional training materials outlining the importance of PROMs collection
McClaery, 2022 [26]	Low enrolment rates, which resulted in staff-assisted patient enrolment support	Issues with visibility and readability, which resulted in the question flow was rearranged to be more logically ordered		Low enrolment rates, which resulted in staff education
Misplon, 2022 [23]	COVID-19, which resulted in the introduction of tele-consultations	A questionnaire based on their own experience with lung cancer care, which resulted in replacing the self-developed questionnaire with an internationally validated instrument		
Neal, 2021 [27]	Supportive services at the cancer centre were not coordinated, which resulted in a coordinated and centralised umbrella organisation being developed, which allowed for increased visibility of available resources to both clinical teams and patients	The frequency of questionnaires was too frequent, which resulted in frequency was changed to every 60 days		
Nordhausen, 2022 [24]		Not all patients appeared in the software program, which resulted in the optimisation of the functionality		
Palos, 2021 [28]		Patients’ lack of knowledge or understanding of the role of PROMs, which resulted in tip sheet was designed to explain the role of PROMs in patient care		Providers lack knowledge of how to assess, interpret, and score the results, which resulted in professional education and training sessions to explain how to use the PROMs and interpret scores
Roberts, 2020 [33]	Patients were concerned that they may miss their doctor’s appointment by stopping to complete the PROMs, which resulted in the touchscreen computer station being located where patients could hear their name called	Difficulties with integrating the report into workflows, which resulted in the alert system being abandoned	No clear process for responding to PROMs symptom information, which resulted in the evaluation of the different pathways for responding to symptom information	The purpose of PROMs not defined and is perceived as just another task added to workloads, which resulted in education about purpose and the evidence for potential uses
Rocque, 2022 [16]	High workload for clinic nurses, which resulted in formalised assignment of survey initiation and compliance to navigators; alert response to clinic nurses	Alert fatigue, which resulted in added a snooze option by setting the expected alert threshold for up to 4 weeks		
Schuler, 2021 [34]		Marked deterioration of PROMs completion, which resulted in scheduling a shared patient-specific PROMs collection task for radiation therapists		Marked deterioration of PROMs completion, which resulted in staff education
Strachna, 2022 [25]		Cognitive load associated with reading patient messages without colours, which resulted in adding colour labels that indicate the level of severity		
Sztankay, 2019 [30]	Lack of privacy (no designated room) in situ, which resulted in completing the PROMs in a separate room	To increase adherence rates, which resulted in adapting assessment frequency to patient preference		
Van Egdom, 2019 [31]		Low compliance rates, which resulted in a brochure to explain the initiative and the different time points of the survey-assessments were created		
Warrington, 2019 [36]		Severe symptom notifications were being triggered for patients reporting retrospective problems, which resulted in a branching question being added to ask patients ‘Is this a current problem?’		Staff found the symptom reports less useful when patients were not completing regularly, which resulted in future training staff being asked to encourage patients to complete regularly and explicitly refer to and use the results in consultations
Wintner, 2020 [32]	Staff did not feel well prepared for PROMs-related critical inquiries of patients, which resulted in phone support for front desk staff	Low response rates, which resulted in refining the cover letter to patients presenting PROMs as a substantial part of the routine practice		
Wu, 2016 [29]		Need more than 1 reminder, which resulted in increasing the frequency of email notifications sent to the patient		

Different dimensions of adaptation for ePROMs and ePREMs—such as context, content, evaluation, and training—address unique barriers and needs; for example, context adaptations involve refining eligibility criteria or clinical workflow, content adaptations change the format, presentation, or symptom highlights, evaluation focuses on readiness surveys and feedback loops, and training covers orientation, role changes, and the practical/technical skill-building required for effective use.

### Common reasons for adaptations

Common reasons for adaptations included a combination of those reported by patients and some reported by clinicians: (i) low response rates and non-completion of ePROMs by patients (*n* = 6) [16, 26, 31, 32, 34, 36], (ii) high workload of clinicians (*n* = 5) [16, 27, 29, 33, 35], (iii) time constraints of clinicians (*n* = 3) [29, 33, 35], (iv) alert fatigue and unwarranted alerts for both (*n* = 5) [16, 25, 29, 36, 37], (v) technical issues for both (*n* = 5) [29, 33, 35–37], (vi) staff’s lack of knowledge about how to assess, interpret, and use the ePROMs system (*n* = 5) [16, 20, 28, 33, 35], and (vii) a high frequency of positive responses that overburdened staff because it resulted in increased assessment (follow-up) for the clinical team (*n* = 1) [27]. Low response rates and non-completion of ePROMs by patients were, for instance, attributed to a combination of infrastructural and human factors, such as a lack of computer access [26], forgetting [36], and patients being unaware of needing to complete ePROMs [34, 35].

To address low response and engagement, possible adaptations included using automated patient reminders [16, 36], refining cover letters or patient training to emphasise the importance of PROMs and the time points of survey assessment [31, 32, 36], providing patients with a tablet device in situ on which to complete ePROMs [26], and staff education (orientations to train new staff and existing staff) [20, 26, 34]. Although some studies provided automated alerts, adaptations included managing those alerts better and making workflow adaptations to address irrelevant and excessive notifications involved. For example, better management of alerts for outpatient staff meant that the staff believed they were not the appropriate person to receive inpatient information when not managing inpatient care [16, 35]. Additionally, making workflow adaptations meant abandoning the alert system in favour of better integration of reports into workflows [33] to address irrelevant and excessive notifications such as revising alert thresholds [20, 36], silencing alerts [16, 25, 37], allowing patients to opt out of receiving calls [16], and adding branching questions to filter current problems [36]. Technical issues, such as connectivity issues [29, 33, 35, 37] and software problems [35], were addressed by adapting the ePROMs system to monitor for and make clinicians aware of connectivity issues [37] and software upgrades [35]. Finally, the staff’s lack of knowledge led to different adaptations, including organising (additional) professional education and training sessions [20, 28], as well as developing detailed guidelines [35].

### Process of integration and adaptations

Various procedures for the integration and adaptation process were reported. Across studies, multiple champions were involved in the adaptation process, including study staff, medical and allied healthcare staff, management and administrative personnel, and information technology experts (Table 4). Twelve (55%) studies used a combination of qualitative (e.g. observation, interviews, focus groups, and written feedback) and quantitative methods (e.g. surveys and outcomes extracted from the EHR) to support and inform the adaptation process, with the majority receiving feedback from the users of ePROMs [16, 19, 20, 26, 30, 32, 33, 35, 36]. The adaptations were collected and presented in different formats: written text (*n* = 11) [21, 24–26, 30–32, 34, 37], a combination of a table and written text (*n* = 6) [16, 22, 28, 29, 33, 36], and supplementary materials (e.g. healthcare provider survey) (*n* = 3) [19, 20, 35].

**Table 4:** Adaptation process

Approach/framework and referenced studies	Description of the integration and adaptation process	Stakeholders involved	Methods and data sources
CFIR (Consolidated Framework for Implementation Research) *Bamgboje-Ayodele, 2022* [20]; *Girgis, 2022* [19]; *McClaery, 2022* [26]	Monthly evaluation and engagement meetings; three pilot studies optimising accessibility	Oncologists, allied professionals, consumer reps, IT/content experts, council, study staff	Quantitative: implementation status, surveys; qualitative: meetings, interviews, focus groups
RE-AIM *Bamgboje-Ayodele, 2022* [20]; *Girgis, 2022* [19]; *Neal, 2021 *[27]	Monthly and iterative feedback in three phases	Multidisciplinary implementation and IT teams, study staff	Quantitative: abstracted outcomes, reports; qualitative: meeting notes, feedback
FRAME (Framework for Modifications and Adaptations) *Rocque, 2022* [16]	Formative evaluation, six steps for adapting implementation in pilot studies	Physicians, nursing, admin, tech vendors, support, study staff	Quantitative: abstracted outcomes; qualitative: verbal feedback, interviews
REP (Replicating Effective Programs) *Sztankay, 2019 *[30]; *Wintner, 2020* [32]	Regular evaluation meetings, continuous feedback and improvement for technical/logistic/workflow issues	Multidisciplinary implementation team, institution reps, medical/therapy directors	Quantitative: surveys, outcomes; qualitative: observations, notes
FITT (Fit between Individuals, Task, and Technology) *Strachna, 2022 *[25]; *Sztankay, 2019* [30]	Regular meetings for feedback collection and process improvement	End users, program managers, clinicians, system developers, work groups	Quantitative: abstracted outcomes, surveys; qualitative: observational notes
iPARIHS (Promoting Action Research in Health Services) *Roberts, 2020* [33]	Five PDSA cycles at prespecified protocol time points	Staff, researchers, patients, study team	Quantitative: abstracted outcomes; qualitative: observation
PDSA (Plan-Do-Study-Act cycles) *Roberts, 2020* [33]; *Wu, 2016* [29]	Five and two cycles, respectively, informing adaptations	Staff, researchers, clinicians, patients, team	Quantitative: abstracted outcomes; qualitative: interviews, observation
MRC (Medical Research Council Framework) *Nordhausen, 2022 *[24]	Three phases with continuous on-site monitoring and team meetings for emerging issues	Study staff	Quantitative: outcomes; qualitative: notes, observation
Usability and Agile Principles *Warrington, 2019* [36]	Iterative testing, feedback collection and integration through regular review	Oncologists, nurses, informatics experts, patient reps, researchers	Quantitative: outcomes, surveys; qualitative: interviews, feedback, observations
User-Centered/Design Principles *Hassett, 2022* [22]	Weekly/monthly meetings to refine design and troubleshoot	Build team, technical leads, site investigators, patients, content experts	Qualitative: user feedback
Action Research/Principles *Fernández-Méndez, 2018* [35]	Iterative cycles of planning, action and monitoring, with regular practical issue discussions	Clinical/admin teams, patients, software developers	Quantitative: outcomes, survey; qualitative: logs
Framework for Implementation Outcomes *Misplon, 2022* [23]	Feedback and workload/validation data collection	Study/clinician team	Quantitative: validation questionnaire, workload, outcomes; qualitative: interviews
No Specific Framework or Approach *Palos, 2021* [28]; Schuler, 2021 [34]; Van Egdom, 2019 [31]	Provider-reported challenges, group solutions, continuous improvement or phased evaluation	Multidisciplinary teams: clinical/administrative, cancer survivors, study authors	Quantitative: outcomes, survey; qualitative: feedback, informal observation
Greenhalgh Integration Feedback *Engelen, 2010 (21*)	Feedback during implementation	Study authors, pediatric oncologists	Qualitative: unstructured feedback
No Specific Approach *Furlong, 2019 *[37]	Monthly evaluation meetings in two phases	Multinational representatives, clinicians, tech partner, researchers	Quantitative: outcomes, feasibility evaluation forms

*FRAME* Framework for Modifications and Adaptations, *REP* replicating effective programmes, *MRC* Medical Research Council framework, *CFIR* Consolidating Framework for Implementation Research, *RE-AIM* Reach, Effectiveness, Adoption, Implementation, and Maintenance framework, *iPARIHS* Promoting Action Research in Health Services framework, *PDSA* Plan-Do-Study-Act cycles, *FITT* Fit between Individuals, Task and Technology framework

### Theoretical frameworks for implementation processes

In *n* = 15 (68%) studies, one (or more) framework(s) or principles were used to guide the development, implementation, adaptation, and/or evaluation [16, 19–27, 29, 30, 33, 35, 36] such as Reach, Effectiveness, Adoption, Implementation, and Maintenance (RE-AIM), Consolidated Framework for Implementation Research (CIFR), and Fit between Individuals, Task and Technology (FITT) (see all listed in Table 4). Some frameworks or principles were specifically used to guide the adaptation process. The frameworks or principles guided the researchers to make adaptations at varying time points in the implementation process, which varied from 3 to 52 months (mean duration = 24.5 months SD 3.2 months). The majority of the studies made adaptations at pre-specified time points (*n* = 11/19), some at random time points (*n* = 7/11), and one included a combination of both (*n* = 1/19). The reason we report out of 19 and not 20 studies is that Bamgboje‐Ayodele [20] and Girgis [19] used the same dataset and were part of the same study. The frameworks were applied in ways that either provided step-wise guidance on how to perform the adaptation process [19, 20, 29, 33, 35] or by guiding the reporting on and categorisation of adaptations [26] (e.g. creating three pilot versions; prioritising adaptations in order of importance). Only one study exclusively reported the use of user-centred design principles [22]. To provide an example of how a framework guided the implementation process, Furlong et al. [37] used RE-AIM framework, which has five domains: (i) reach of the target population, (ii) effect on key outcomes, (iii) adoption by people responsible for its delivery, (iv) success of its implementation, and (v) potential for it to be maintained. Each of these five domains includes specific questions that researchers or implementers can ask or address, using data sources (e.g., clinical audit logs completed over 3 months, EHR data, and ePROM survey) and at different time points. This is applied arbitrarily or as decided by the particular team. There was no consistency or pattern in terms of the framework used and phases or frequency of data collection, stakeholders involved, access location, ePROMs measurement use, or frequency or types of adaptations made.

## Discussion

### Principal findings

This review summarises the type of adaptations made to ePROMs during implementation using data from *n* = 20 studies. Various data collection methods and stakeholders were utilised to make adaptations, guided by one or more implementation frameworks (80% of studies). Common types of adaptations included changing context (e.g. complex onboarding), content (e.g. readability) (all studies), evaluation (e.g. alerts), and training of patients and clinicians (e.g. training). No evidence was found for the type of implementation framework or the number of types of adaptations made. Overall, the existing evidence of ePROMs adaptations made during implementation in clinical care in the published literature is rather limited, and although the studies contain varying degrees of details of the adaptations made, there is no standardised method for reporting these adaptations or assessing their efficacy.

At the time of writing this review, this was the first review to date on the topic of how studies undertake and report on adaptations of ePROMs/ePREMs systems for integration into oncology care. In addition, there are no reports on the reasons and types of adaptations made to system design and/or implementation strategy to promote implementation. Similar reviews exist on the implementation of ePROMs systems in general [2, 8, 39, 42], but not explicitly on the adaptations made during process implementation, which is a unique contribution of this review to the literature gap. One of the recent reviews also shows similar findings related to the adaptations [39]. For example, the review demonstrated that adaptations require personalisation and are often not aligned with clinical reality, calling for the development of ePROMs that would fit better with the existing workflows and programmes [39]. We found no evidence that an application of a particular implementation framework results in capturing or applying a different number or types of adaptations to any of the dimensions: content, context, evaluation, or training. The data also show no consistency in terms of the implementation framework that guided the implementation process and all other factors such as phases or frequency of data collection, stakeholders involved, access location, choice of ePROMs, or frequency or types of adaptations.

### What this study adds and implications for practice

We did not aim to report on the successes or failures of the implementations and adaptations of ePROMs in clinical practice. Instead, we attempted to map which types of adaptations are made within a local context, and how they are reported and captured during the implementation of each study. Each study had made sets of adaptations specific to their local context, but we could not identify any patterns apart from that they have all ended up making changes to the content (e.g. changing visibility or presentation, readability, and feedback for users within the ePROMs system) of ePROMs during implementation, and that all included various stakeholders in the adaptation process. The majority of the changes were therefore to the presentation, but also to alerts, response options, and frequency of administration of ePROMs. We did not collect data on whether the changes improved ePROMs response rates because this was not the objective of the review. It is possible that the complexity of changes was likely individual to every context, cancer group, ePROM, and individual study, which would warrant further review in future. The studies included a large variety of stakeholders in the adaptation process, ranging from oncologists, nursing and allied staff, clinical and administrative teams, technology partners, study staff, study authors, health informatics experts, researchers, advisory workgroups, etc. Although this was not our aim, we became aware that the studies do not describe which method or group of stakeholders was more or less effective in making adaptations and when, because this was entirely individual to each study, their target clinical and patient population, their chosen ePROMs system, the chosen implementation framework(s) and their local contexts [11].

The studies included in this review have captured the adaptations in various ways, using a structured and unstructured collection of feedback; through mostly qualitative but also quantitative feedback, such as (semi-structured) interviews, focus groups, reports, technological feasibility evaluation forms, observation notes, meeting logs, evaluation of transcripts at engagement meetings and detailed notes, and abstracted outcomes. Mixed methods are a preferred method to capture data in this context; however, it was not an objective of this review to evaluate the levels of organisational readiness for implementing change, nor the effect the clinicians had on the success or use of ePROMs [19, 43].

We could not assess the clinical impact of the adaptations made as has been done in some reviews [44] because this was not the objective of this review but also because many studies did not report the outcomes of the adaptations but just the adaptations made (usually as a list or within the text). However, we succeeded in offering a comprehensive, detailed (up to May 5, 2023, for databases and July 24, 2023, for grey literature) description of types and reasons for adaptations of oncology ePROMs during implementation—which should be of great value to any implementation scientists wishing to develop, evaluate, or implement PROMs into clinical practice across different clinical specialties.

There were no studies found that detailed the adaptation of ePREMs in routine cancer care. The reasons for this are unknown; however, it could possibly be due to differences in how ePROMs versus ePREMs are used and/or studied. Future research should focus on the use of ePREMs as well as ePROMs more, particularly how they are adapted for local contexts. Also, this review identified the limited data on the use of ePROMs in in-patients, and these groups could benefit from being included more frequently in this literature. This could point to a broader problem of not (yet) including people’s *experience* measures (ePREMs) in care as frequently as their medical *outcomes* (ePROMs) [45].

### Strengths and limitations of the study

The strengths of this review include compliance with PRISMA-ScR guidelines, the development of a comprehensive search strategy, and the review of results applying the PCC framework [14]. The results of the review help further the conversation on ePROMs implementation needs in oncological clinical practice and shed light on the interaction between people and technology in a real-world clinical setting. A closer inspection is required to elucidate the interplay between patient management based on ePROMs results and the success of care. The findings of this review are likely transferable and generalisable to other ePROMs in medical fields, which include other patient populations and countries. This is because digital ePROMs are digital tools, and digital tools of a certain nature often share similar characteristics. For example, ePROMs are designed with a specific purpose of gathering patient information for more streamlined and patient-centred care. The issue with patient-centred data is that it is not always collected efficiently, in a cost-effective manner or frequently enough. Introducing ways to improve this process, i.e. by adapting the implementation and use of ePROMs, may yield increased efficiency, better response rates, and data quality, which in turn enhances clinical decision-making or enables better identification of patients’ health changes or health needs. Since all ePROMs are designed with this purpose and implemented into various clinical fields, they likely share similar challenges.

The review also has some limitations, including the relatively small number of databases and papers included in the review. The grey literature search resulted in adding one paper which was published in the English language. Grey literature from other languages could potentially result in capturing more studies and more insights or details of adaptations made during the implementation of ePROMs as part of the routine oncology care. Another limitation of this review is that it was not possible to capture or describe each of the implementation frameworks used in each study due to the sheer size and aspects of this information. A separate review could be carried out in describing just the implementation of different frameworks used in ePROMs in oncology.

Clearly, we could only examine the material available to us. We did not investigate the effectiveness or outcomes of the adaptations, but rather focused on identifying and reporting them. Our aim was to understand the nature of the adaptations, not their impact (e.g. in practice or for oncology populations), as this was beyond the scope of our study. The general trend we observed was that adaptations made to ePROMs during the implementation are highly contextualised to the local setting in which they are implemented. This observation aligns with findings from other studies [2].

Studies have also pointed to the importance of clinicians in implementing systematic ePROMs/ePREMs, with increased engagement from clinicians leading to higher success rates in patient enrolment and responses [40]. Our review did not assess the clinicians’ involvement, be it to elicit patients’ feedback or as part of the adaptation process. Not describing clinicians’ involvement with ePROMs/ePREMs as part of adaptations more broadly can mean that we have not considered adaptations that happen on a more macro level [41, 46].

Lastly, this review had several methodological limitations. We could not capture the function of PROMs at different levels (e.g. PROMs as a screening tool, a management tool to identify and prioritise issues, or a tool to improve patient-physician information [41, 47]). Additionally, our search of literature could have been widened, and findings of ePROMs adaptations could have been compared based on the different conditions, such as cancer and non-cancer diseases, for example. We could not assess if specific feedback and later types of adaptations were linked to different functions of PROMs, nor if different implementation frameworks yielded different adaptation types. Also, our exclusion criteria included studies that exclusively focus on looking at the effectiveness of ePROMs/PREMs, but details of adaptations may have been included in such studies. We also recognise that the review was conducted in a specific timeframe and data extracted by specific researchers and as such, it is apparent that any potentially relevant new literature published afterwards will not have been included. This time lag may limit the comprehensiveness of the most-up-to-date evidence.

## Conclusion

To our knowledge, this was the first scoping review to explicitly describe and categorise adaptations made to ePROMs in oncology during digital implementation. Previous and existing reviews briefly mention adaptations, but do not describe them in detail. Although an increasing amount of literature continues to be published on the implementation of ePROMs systems in oncology care worldwide, several “grey areas” remain. These include systematically reporting and managing feedback from patients and clinicians, as well as measuring the effectiveness of adaptations. This review may broadly suggest that the adaptations are dependent on local needs and structures, and what works for whom and when. The findings of this review demonstrate that adaptations during an implementation process for ePROMs in oncology happen by using phased approaches, holding regular meetings with stakeholders, continuous testing and monitoring of the tool and feedback, using multiple clinical champions, and not relying solely on the quantitative data but instead relying more heavily on qualitative reports of immediate users. Future research could focus on describing factors for the overall success of an adaptation (i.e. better outcomes).

## Supplementary Information

Below is the link to the electronic supplementary material.

ESM 1 (DOCX 17.6 KB)

## Data Availability

Further information about the search strategies and data extraction are available from the corresponding author on reasonable request.
