# Supplementary material for: Adaptation of digital integration of PROMs and PREMs in oncology during implementation: a scoping review
Source: Support Care Cancer. 2026 Mar 13;34(4):316. doi: 10.1007/s00520-026-10509-0 (PMC12987908; doi:10.1007/s00520-026-10509-0)
Supplement: Supplementary file 1 — (DOCX 17.6 KB) [file 520_2026_10509_MOESM1_ESM.docx]

**Supplementary material 01**

Supplementary Table 1: Search Terms and the inclusion and exclusion criteria

| **Research aim/question:** |  |
| --- | --- |
| This scoping review aims to provide insight into the implementation, adoption and adaptation processes of digital PROMs and PREMs in oncology care. | |
| The specific research questions are: | |
| i) how studies report on adaptations during an implementation process of ePROMs/ePREMs systems in cancer care; | |
| ii) reasons and types of adaptations for implementation. | |
| **Population, Concept, and Context (PCC)/Population, Interest, and Context (PICo) - checklist framework:** | |
| Population: oncology |  |
| Concept/interest: the implementation, adoption, **and** adaptation process of digital PROMs and/or PREMs | |
| Context: oncology centres/institutes/departments | |
| **Search string:** | (see **Supplementary Material 2**) |
| **Checklist:** |  |
|  | **Criteria** |
| **Population** | Inclusion: oncology patients & relevant stakeholders Exclusion: NA |
| **Concept/interest** | Inclusion: **digital PROMs and/or PREMs** Exclusion: paper-based PROMs and/or PREMs |
| **Concept/interest** | Inclusion: description of the **implementation, adoption and adaptation proces** (Information on/assessing adaptations/strategies/tactics/modifications etc.? Decisions made to adapt? Troubleshoot? Iterations?) Exclusion: studies exclusively focused on the impact, effectiveness, measurement properties, language validation; comparison of treatments studies; PROMs used as a measure of cancer therapy effect; the implementation of ePROMs and/or ePREMs in research contexts (e.g., clinical trials) |
| **Context** | Inclusion: oncology centres/institutes/departments  Exclusion: studies that deal with other settings not related to oncology |
| **Sources of evidence** | Inclusion: English and Dutch (grey literature) Exclusion: non-English language |
| **Sources of evidence** | Inclusion: **original research** Exclusion: duplicates, published literature other than primary studies such as protocols, nonempirical publications, editorials, letters to editors, comments to other publications, conference proceedings, and reviews; no further restrictions are made on the study design |
| We focus on studies applying an iterative approach given the iterative nature of adaptation processes for integration into standard of care | |
|  |  |
| **Definitions:** |  |
| **Adaptation** | Specific, planned, and purposeful changes to the intervention that enhance acceptability and fit to a local context |
| **Implementation** | The process of introducing a new system within an organisation (from planning through to routine use) |
| **Adoption** | The process of starting to use a new technology either on an individual or a group level |
| **Appropriation** | An early encounter between a new ICT and a household, or an individual |
| **Stirman and associates’ typology of modifications:** | |
| **Context** | Modifications to the format or channel, the setting or location in which the overall intervention is delivered, or the personnel who deliver the intervention |
| **Content** | Content modifications are changes made to the inter-vention procedures, materials or delivery |
| **Training and evaluation** | Changes made to the procedures for training personnel or evaluating the program |
|  |  |
| **References for the definitions** | 1. BMJ 2021;374:n1679  2. Wiltsey Stirman et al. Implementation Science (2019) 14:58. https://doi.org/10.1186/s13012-019-0898-y 3. van Gemert-Pijnen J. L. (2022). Implementation of health technology: Directions for research and practice. Frontiers in digital health, 4, 1030194. https://doi.org/10.3389/fdgth.2022.1030194 4. Phichitchaisopa, N., & Naenna, T. (2013). Factors affecting the adoption of healthcare information technology. EXCLI journal, 12, 413–436. |
